# Experimental Verification of High-Temperature Resistance and High Resolution of Inductive Tip Clearance Measurement System

**DOI:** 10.3390/s25103145

**Published:** 2025-05-16

**Authors:** Ziyu Zhao, Lingqiang Zhao, Yaguo Lyu, Zhenxia Liu

**Affiliations:** 1 School of Missile Engineering, Rocket Force University of Engineering, Xi’an 710025, China; 2 School of Power and Energy, Northwestern Polytechnical University, Xi’an 710129, China; lingqiangzhao2018@mail.nwpu.edu.cn (L.Z.); yglu@nwpu.edu.cn (Y.L.); zxliu@nwpu.edu.cn (Z.L.)

**Keywords:** inductive sensor, high temperature, dynamic tip clearance, high resolution

## Abstract

An inductive clearance measurement sensor has advantages of good anti-interference, fast response speed, and high sensitivity, and it has obvious technical potential in aeroengine turbine tip clearance measurement. In this paper, a rotor dynamic tip clearance measurement experiment system was designed based on a high-resolution inductive measurement system. The high temperature calibration experiment, performance verification experiment, and dynamic clearance measurement experiment under varying operating conditions were used to verify the high-temperature dynamic measurement performance of the measurement system. The resolution was used as the evaluation parameter of measurement performance. The experimental result shows the system has good resolution and dynamic response at 1000 °C, and the dynamic resolution reaches 10 μm in the 3 mm measuring range. The varying condition experiment results show that the blade deformation caused by the speed change of 1000–3000 r/min and the temperature change of 600–1000 °C can be resolved, and the resolution reaches about 10 μm. The research results verify that the inductance clearance measurement system has the characteristics of high temperature resistance and high resolution, and the technical specifications of clearance detection meet the basic requirements of dynamic clearance measurement of turbine tips, which provides an effective detection method for aeroengine health monitoring.

## 1. Introduction

As the core component of aeroengines, high-pressure turbine performance has a decisive impact on engine performance, while the abnormal tip clearance of a turbine is the main factor causing performance degradation. Small tip clearance can reduce the amount of gas clearance leakage and improve turbine efficiency, as while engine efficiency. However, too small of a tip clearance may cause the blades to collide with the casing during the acceleration and deceleration process, which will cause excessive engine vibration at minimum or lead to blade fracture in severe cases, consequently endangering the operational safety of the engine [1]. Therefore, accurate design or active control of turbine tip clearance is necessary to improve engine performance and ensure operation safety. For this purpose, accurate real-time measurement of tip clearance is required.

The designed value of tip clearance of high-pressure turbines of a certain engine is about 2.7 mm [2], and the maximum variation in clearance is about 1.5 mm [3]. In addition, the high temperature, high speed, and corrosive gas environment of turbines impose strict requirements on the temperature resistance, measuring range, resolution, dynamic response, and corrosion protection of the sensor probes. At the same time, due to the compact structure of turbine components, the structure and installation of the probes have strict requirements. At present, the main measurement methods for turbine tip clearance include the microwave method, optical method, capacitance method, and inductance method.

The microwave method has been used to measure the dynamic clearance of a rotor tip at 3000 r/min, and the clearance measurement accuracy was better than 40 μm in the range of 0.5–3 mm [4]. The products of Meggitt Company (Villars-sur-Glâne, Switzerland) were applied in the structural health monitoring and active clearance control of aeroengines. The probes can withstand temperatures up to 900 °C without cooling and 1200 °C with cooling, and the measurement accuracy achieved 25 μm [5].

In 2009, Vakhtin et al. [6] from Southwest Technologies Inc. (North Kansas, MO, US) designed a co-path interferometric sensor based on sapphire fiber and sapphire window. The probe diameter was 12.7 mm, with a temperature resistance of 600 °C. It could complete a single measurement in approximately 20 μs, with a measurement range of 0.9 mm and better than 10 μm accuracy. The inclined dual-fiber bundle sensor of Hood Technology (Cambridge, MA, US) can withstand a temperature of 650 °C without a cooling device and 1100 °C with cooling [7]. The measurement error of blade tip clearance at a 549–1365 r/min rotation speed by the laser optical method was about 30 μm, and the speed measurement error was 1% [8].

The dynamic gap measurement results by the capacitance method in the literature [9] indicated the gap measurement error was less than 30 μm when speed was lower than 1000 r/min, while the error was about 65 μm at 2500 r/min. In 2020, Satish et al. [10] from the Gas Turbine Research Institute of India proposed a novel conditioning circuit with a bandwidth of 700 kHz and completed the tip clearance test on the second-stage fan blades of aeroengines; with a measurement range of 0.4–3 mm, resolution was better than 2.5 μm. The active cooling capacitive sensor developed by Rotadata Ltd. (Derby, UK) can withstand temperatures of 1400 °C and 1000 °C without cooling [11]. The products developed by Fogale Company (Nimes, France) are the most widely used. Its MC925 system has a 230 kHz bandwidth, 3 mm measurement range, and the sensor can withstand a temperature of 1400 °C with cooling [12].

A comparison of the various measurement methods is given in Table 1.

Considering the optical sensor is easily interfered with by impurities in the gas, it has strict requirements for measurement environment clarity; thus, it is not suitable for long-term measurement in the engine environment. The capacitive sensor is greatly affected by the dielectric constant of the environment; there are zero drift and breakdown problems, and it also requires a fine sensor calibration system. In comparison, inductive sensors have advantages of long-term high-temperature stability, a simple structure and versatility, and the obvious potential in the application of turbine tip clearance measurement.

In the 1990s, the NASA Lewis Research Center developed an inductive turbine tip clearance measurement device, and the sensor had a temperature resistance about 400 °C [13]. K. S. Chana et al. from the University of Oxford [14,15] began to use inductive sensors to test blade tip clearance on gas turbines in 2008. A series of published research results showed that the developed sensor has high repeatability for the gap measurement of different blade tip structures, but the sensor output signal is affected by temperature changes when the temperature is above 1075 K [14]. The improved sensor can be used at high temperatures up to 1400 °C, and the test results on a high-speed rotor showed that the dynamic response of the sensor meets the measurement of turbine tip clearance at a speed of 68,000 r/min [15]. Jiang Zhe et al. [16,17] from Akron University have conducted in-depth studies on the measurement accuracy and temperature impact analysis of inductive sensors. The research results published in 2014 showed that the displacement resolution of an inductive sensor based on a planar hollow coil is better than 10 μm, and the dynamic gap measurement at 8000 r/min proved the sensor dynamic response [16]. Subsequently, experimental results on the temperature sensitivity of inductive sensors showed that temperature had a significant impact on the calibration results of sensor characteristics within 700–1300 K [17]. Edward Rokicki et al. [18] proved inductive sensors have good high-temperature resistance performance at 1000 °C by a heating test. The studies above have provided significant guidance in the development and research of high-temperature resistant eddy current tip clearance testing systems. Cui D. W. et al. [19] determined coil shape and material through electromagnetic simulation and mechano-thermal simulation, and they designed a three-dimensional spiral high-temperature probe composed of a low-temperature co-fired ceramic substrate and a Ag coil, with a temperature resistance of 600 °C. Sergey Borovik et al. [20,21] developed a method to monitor the states of gas turbine engines based on single-coil eddy current sensors, which can withstand temperatures up to 1200 °C, and they provided an overview of the application adaptability issues of inductive measurement systems on high-temperature components of engine turbines, including the main affecting factors on measurement reliability and accuracy such as the complex blade shape surfaces, the multidimensional movement of the structures, and restrictions on the number of probe and their installation.

In the research on inductive clearance measurement system characteristics, Yang Q. et al. [22] measured the axial clearance of a scroll compressor by an inductive method. The sensor measurement range was 0.05 mm to 0.55 mm, and the dynamic resolution was 0.5 μm. Li, H. et al. [23] designed several radial sensors with different gaps and coil turns, and the influence of gap and coil turns on the sensor output voltage and sensitivity was studied through finite element simulation and experiments. Wang W. M. et al. [24] proposed a measurement method for blade clearance and arrival time based on a trigger pulse using an inductive method. Experimental results proved the system measurement accuracy was less than 60 μm, and the response frequency was up to 10 kHz. Ye D. C. et al. [25] proposed a crowd blade vibration measurement method based on a high-frequency response inductive sensor. A signal processing circuit with 250 kHz bandwidth met the dynamic response requirement at a 3000 r/min rotation speed. Zhao L. Q. et al. [26] considered the spatial filtering effect of high-speed rotating blades and conducted an experimental study on the influence of planar coil shape and detection target structure on the performance of inductive clearance measurement systems. Sillanpää T. et al. [27] proposed a robust and compact three-dimensional position sensor that can measure the rotor displacement in both the radial and axial directions, and they verified this in a 15,000 r/min high-speed industrial induction machine. The above studies were conducted at normal temperature.

In summary, scholars have carried out extensive theoretical and experimental studies on turbine tip clearance measurement methods, as well as clearance measurement performance verification and optimization, which promoted the development of inductive measurement technology. However, there is lack of performance verification studies of the inductive methods at high temperature (over 500 °C), especially application verification research for the engine turbine conditions. Therefore, based on the previously developed high-temperature sensor probe, high-temperature calibration system, and high-temperature rotor dynamic test setup [28], this paper carried out high-temperature dynamic tip clearance measurement tests to verify that the measurement range, dynamic resolution, response speed, and other performance indicators and functions of the inductive system met the requirements of turbine dynamic tip clearance measurement. The research in this paper proves the effectiveness of the inductive method in engine structure health monitoring, and it promotes technology to test engines in extreme environments.

## 2. High-Temperature Verification of the System

### 2.1. Measurement Principle and Calibration Method

**Figure 1:**
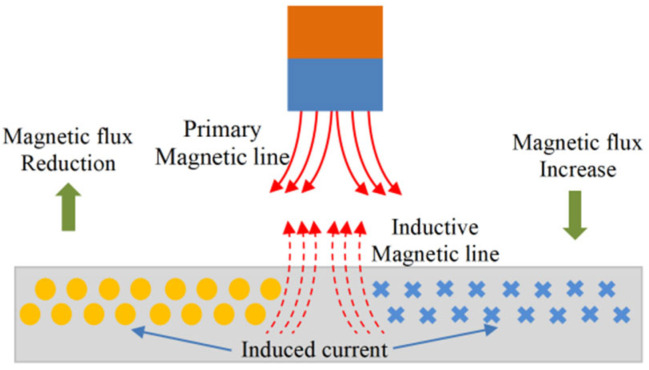
Principle of Lenz’s law.

The principle of the inductive sensor is based on Faraday’s law of electromagnetic induction and Lenz’s law. The main component of the sensor is an inductive coil, which can be a three-dimensional spiral coil or a two-dimensional planar coil. When the coil is excited by a high frequency AC signal, it generates a magnetic field. As a metallic object passes through this magnetic field, eddy currents are induced within the object material. These eddy currents, in turn, generate a reverse magnetic flux, which acts to reduce the inductance of the sensor coil. Figure 1 shows the working principle of the inductive clearance sensor.

According to Lenz’s law, there is a certain relationship between the coil equivalent inductance and the detection gap, that is, the gap detection characteristics of the sensor. When the coil structure and the excitation signal are determined, there is a univariate functional relationship between coil inductance *L*_C_ and the detection gap *d*: $L_{c}=L_{c}(d)$. Therefore, under the coil structure and excitation signal-determined condition, the coil equivalent inductance of the measuring coil changes with gap *d*, and there is a single corresponding relationship. Therefore, the corresponding relationship between coil inductance and clearance is obtained by calibration before the measurement, that is, the sensor characteristic curve.

**Figure 2:**
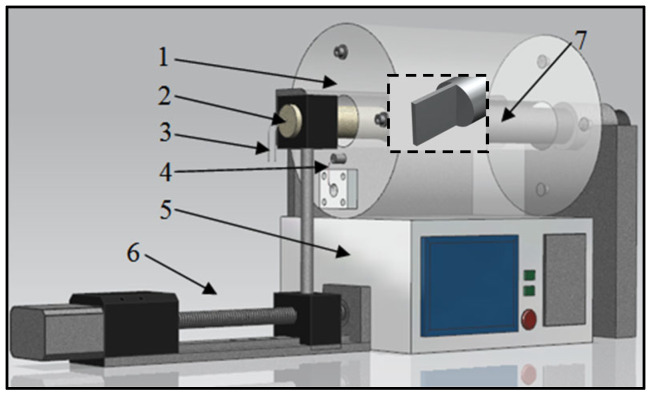
Calibration system at high temperature.

Due to the inductive clearance measuring principle, the parameters of the inductive sensor are greatly affected by the ambient temperature, so it is necessary to use a calibration process at the same temperature as the actual gap measurement ambient temperature. The high-temperature calibration system, as Figure 2 shows, was adopted in this study.

The system consists of a cylindrical heater (1) with a 1200 °C maximum heating temperature and 1 °C temperature control precision by a thermal couple (4) and PID controller (5), a high-precision position adjuster with ±1 μm accuracy which is controlled through the motor driving screw (6), a sensor probe which has a coil placed inside the heater (2) and external connecting wire (3), and a metal object which simulates the blade (7). For easier installation, the calibration object was processed into a metal rod with 10 mm diameter and 30 cm length. One end of the rod was cut into a thin slice with 15 mm height and 1.5 mm thickness, which was equivalent to the actual turbine blade thickness. The probe and the object are exposed to the same high-temperature environment and the distance between them could be adjusted; therefore, the sensor signals at different temperatures and clearances can be obtained by this setup.

**Figure 3:**
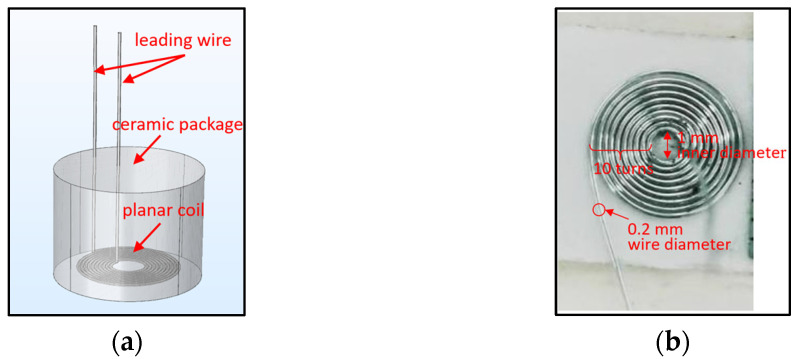
(**a**) Sensor probe structure; (**b**) planar coil structure.

Figure 3 shows the sensor probe structure. The main element was a planar coil made of platinum wire with a melting point over 2273 °C, and it was enclosed in a high-temperature ceramic adhesive gel, which kept the coil geometry stable at high temperatures and protected the coil from corrosion.

The maximum temperature of the sensor proposed in this paper reached 1000 °C. In order to adapt to the oxidation environment at high temperatures, the calibration target material should have similar performance to the actual blade material and should also meet the requirements of high-temperature resistance, oxidation resistance, and corrosion resistance. Table 2 lists four types of high-temperature nickel-based alloy materials commonly used in gas turbine blades, which have sufficient strength and creep resistance. By comparing the material characteristics and considering the requirements of the experiment’s environment, the target material was determined as Inconel718 (US grade).

**Figure 4:**
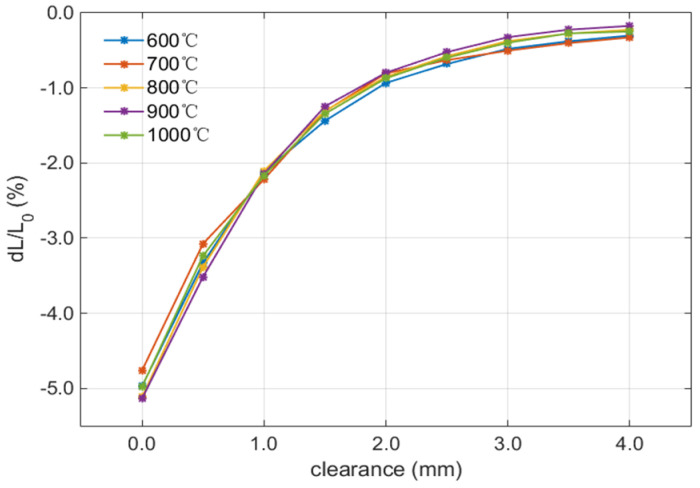
High−temperature calibration curve of sensor.

By adjusting the heating temperature, static calibration experiments were carried out at 600 °C, 700 °C, 800 °C, 900 °C, and 1000 °C, respectively. The relative variation in sensor inductance (d*L*/*L*_0_) was taken as the calibration parameter, and the clearance between the sensor probe and the target surface was adjusted by the displacement controller. The characteristic curves in the range of 0–4 mm, as shown in Figure 4, were obtained with a 0.5 mm interval. The fitting curves at different temperatures have good coincidence and the same characteristics within the range of 0–4 mm. The calibration parameter (d*L*/*L*_0_) changed from about −5% to about 0% within the full measurement range, while the sensitivity of the calibration parameter decreases gradually with the gap change. Within the range of 0–1 mm, d*L*/*L*_0_ sensitivity was close to 3%/mm, while it was over 1%/mm, 0.5%/mm, and 0.2%/mm within 1–2 mm, 2–3 mm, and 3–4 mm, respectively, indicating the sensor sensitivity is lower at the large gap.

In the subsequent high-temperature dynamic measurement experiment, the calibration data were input data in the data process to calculate the gap value based on the measured d*L*/*L*_0_.

### 2.2. High-Temperature Dynamic Experiment

**Figure 5:**
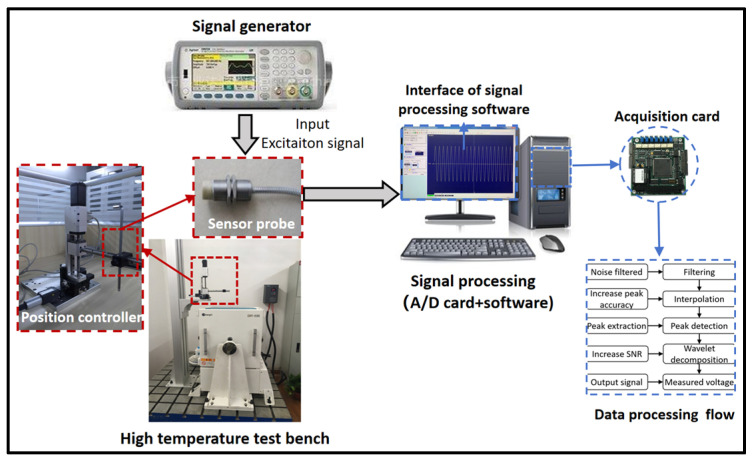
High-temperature tip clearance measurement system.

The experimental system for the dynamic clearance measurement under high temperatures included the rotor component, heating and temperature control system, rotor drive system, brace system, and clearance measurement system, and the main functions simulated a high-temperature environment, rotor driving component, and fixing and adjusting the distance between the sensor and target. According to the function, the experiment system was divided into two parts, comprising a high-temperature mechanical system and a measurement system (Figure 5).

#### 2.2.1. High-Temperature Mechanical System

**Figure 6:**
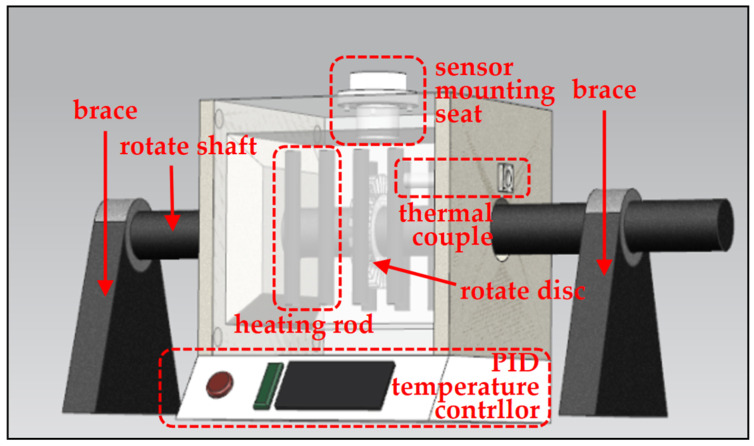
Perspective structure of test bench.

The high-temperature mechanical system included a high-temperature control system, rotor test component, rotor driving motor, and brace system. Figure 6 shows the perspective structure of the high-temperature dynamic tip clearance measurement test bench.

The high-temperature control system of the test bench provided a stable high-temperature environment for measurement. It was composed of a box heating furnace and a digital temperature controller. The rotate shaft ran through the furnace by punching two symmetrical holes on the left and right walls. The gap measurement sensor mounting seat used to fix the eddy current tip gap measurement sensor was installed on the top wall with a through-hole above the rotor. The inner dimension of the furnace was 120 mm × 120 mm × 120 mm. The PID temperature controller was under the furnace, and the thermocouple installed on the furnace side wall was used to monitor the heating temperature; thus, the heating process could be customized and monitored. Table 3 lists the performance parameters of the heating furnace.

**Figure 7:**
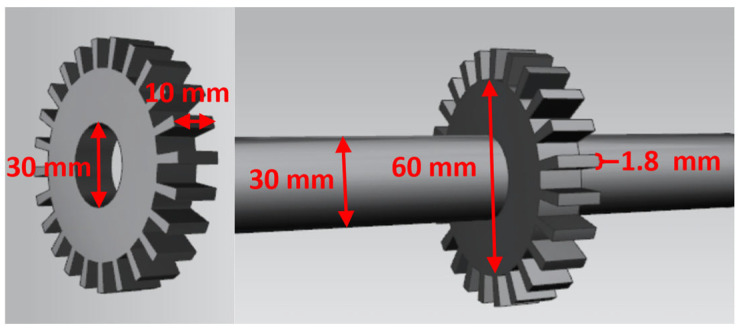
Structure of test rotor.

Figure 7 shows the structure of the rotor component. It was a round disk with a 60 mm outer diameter, 30 mm inner diameter, and 10 mm thickness. There were 24 uniformly distributed blades with a 10 mm height and 1.8 mm blade tip width. The rotate shaft diameter was 30 mm and had a length of 815 mm. The rotor disk was connected with the upper convex of the rotating shaft by screws.

**Figure 8:**
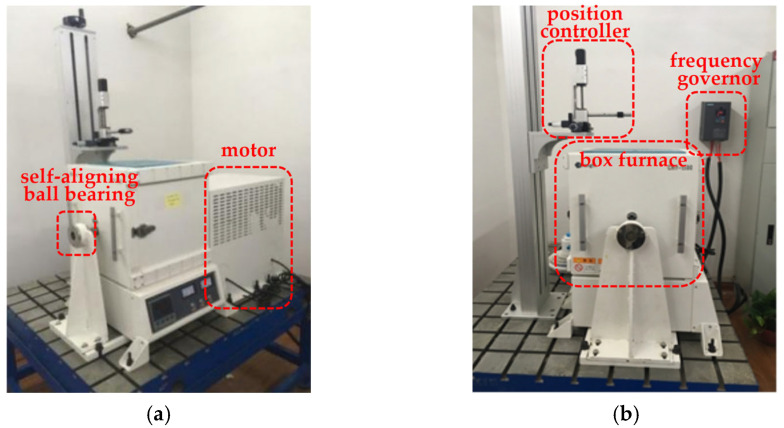
High-temperature dynamic blade tip clearance measurement test bench: (**a**) main view; (**b**) side view.

The rotor drive system adopted a high-speed variable frequency motor, and the speed could be adjusted in the range of 1000–15,000 r /min through the variable frequency governor. The motor shaft and the drive shaft were connected through diaphragm coupling, which compensated for the relative displacement of the axes of two shafts, as well as the buffer and anti-vibration. The rotor support adopted extended external support with two pivots, and the bearings were, respectively, a self-aligning ball bearing and cylindrical roller bearing (Figure 8).

#### 2.2.2. Sensing and Measurement System

The inductive gap measurement system mainly included the inductive sensor probe, measurement circuit, signal generator, high-speed data acquisition system, and acquisition signal processing software; the main function of the system was to achieve the rotor dynamic tip clearance measurement, data processing, and result output.

**Figure 9:**
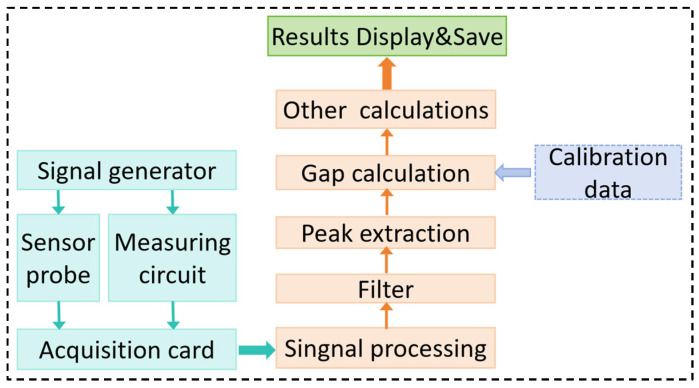
Measurement signal generating and processing.

Figure 9 shows the gap measurement signal processing process. The signal generator provided the standard excitation voltage for the sensor and measurement circuit. The output signals of the sensor and the circuit were converted to A/D through the data acquisition card and then input to the computer for data processing and analysis. The acquisition signal processing program and analysis functions included sensor calibration characteristic data entry, original signal filtering, sinusoidal signal peak extraction, gap value calculation, and measurement result output and saving. The program was developed in Matlab 2014.

Based on the performance of the high-temperature mechanical system and measurement system equipment, the main parameters of the high-temperature dynamic measurement platform are listed in Table 4.

#### 2.2.3. High-Temperature Dynamic Experiment Conditions

In order to verify the high-temperature dynamic measurement performance of the inductive measurement system, the experiments were carried out according to the operating parameters listed in Table 5. The system vibration was small enough when the rotation speed was lower than 3000 r/min, it could be considered that the gap change was caused by the structure deformation caused by thermal load and centrifugal load. Therefore, the rotor speed was controlled below 3000 r/min in the dynamic experiment to avoid the influence of excessive vibration on precise dynamic measurement results.

The ambient temperature in the turbine tip area was determined by the temperature distribution of the combustion chamber outlet, leakage gas temperature, and cooling air temperature. In the cycle condition of a certain engine, the gas temperature was from 700 °C to 1200 °C, considering the uneven temperature distribution of the combustion chamber outlet and the cooling air mix, as well as the temperature resistance of the sensor probe; the temperature condition was set to 600–1000 °C in the dynamic experiment.

In the resolution verification experiment, the installation gap was determined as 3 mm, which was the same as the sensor measuring range to be verified. The dynamic resolution of the measurement system in the full range could be verified by the measured dynamic gap distribution of the entire rotor. Then, the gap distributions of the entire rotor at variable speed and variable temperature were determined, respectively, to verify the capability of the measurement system to detect the influence of rotation speed (centrifugal load) and temperature (thermal load) on the gap variation.

## 3. Results

### 3.1. Dynamic Resolution Verification

The dynamic resolution verification experiment was carried out at 1000 °C and 3000 r/min. The measurement system continuously obtained the original signal of the whole rotor passing through the sensor and the signal of each blade passing through, obtained by filtering and peak extraction process. Then, it calculated each blade tip clearance through the calculation and interpolation method based on the calibration curve.

**Figure 10:**
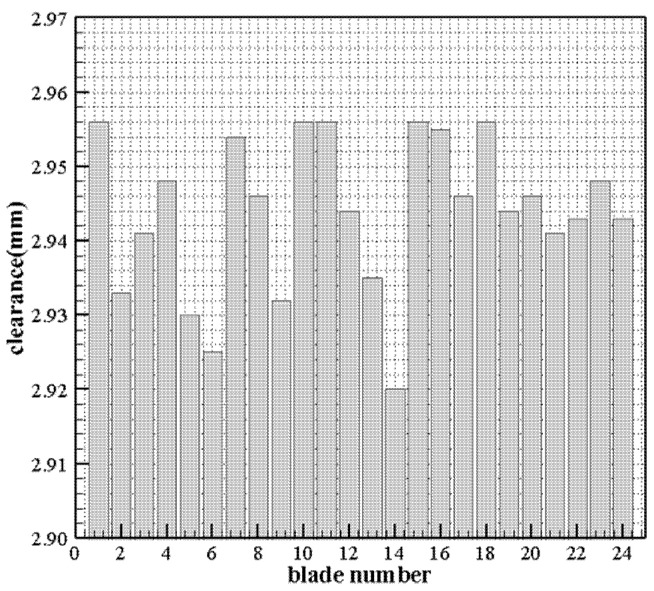
Blade tip clearance measurement result at 1000 °C.

Figure 10 shows the dynamic measurement results of the rotor tip clearance throughout the rotor cycle, and Table 6 lists the measured values of each blade tip clearance. It can be seen from the figure and data that 1. the maximum value, minimum value, and the range of blade tip clearances of the whole rotor at experimental conditions are 2.956 mm, 2.920 mm, and 0.036 mm, respectively. 2. Comparing the tip clearances and the longitudinal interval (10 μm), it can be found the dynamic resolution of the measurement system for the rotating blade is higher than 10 μm at the 3.0 mm installation gap; furthermore, it can be inferred that the dynamic resolution of the measurement system is higher than 10 μm according to the “near high and far low” resolution change characteristics of the inductive sensor from the calibration curve.

### 3.2. Dynamic Measurement Results at Variable Conditions

#### 3.2.1. Dynamic Results at Variable Speed Conditions

**Figure 11:**
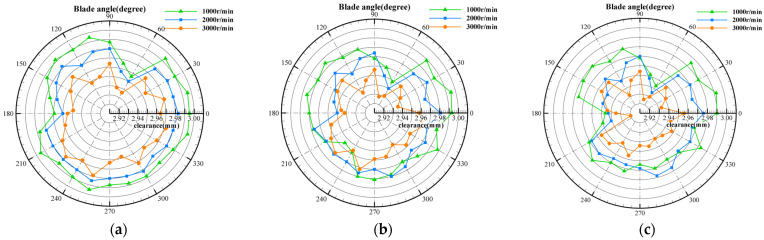
Measurement result of blade tip clearance at different rotation speeds: (**a**) at 600 °C; (**b**) at 800 °C; (**c**) at 1000 °C.

In order to verify the dynamic measurement ability of the measurement system for blade tip clearance at different rotation speeds, the same experimental method as shown in the previous section was adopted to obtain the measurement results of the whole rotor blade tip clearance when the sensor installation gap was 3 mm and the rotation speed was 1000 r/min, 2000 r/min, and 3000 r/min, respectively. The results are illustrated in Figure 11a–c.

The following can be seen from Figure 11:

(1) The tip clearance varies significantly with the speed at different temperatures. The centrifugal load increases with the speed increasing, and the clearance value decreases obviously throughout the whole rotor.
(2) Compared with Figure 11a–c, the thermal load increases as the temperature rises, and the clearance value decreases in the whole rotor.

The dynamic results corresponding to different rotation speeds at different temperatures are listed in Table 7. The rotor average clearance variations with the rotation speed increasing from 1000 r/min to 3000 r/min are 0.026 mm, 0.025 mm, and 0.027 mm, respectively, when the temperature is 600 °C, 800 °C, and 1000 °C. It proves that the blade deformation caused by the centrifugal load is similar under different temperature conditions, and the dynamic resolution is proved up to 10 μm by comparing the measured clearance values at different rotation speeds.

#### 3.2.2. Dynamic Results at Variable Temperature Conditions

**Figure 12:**
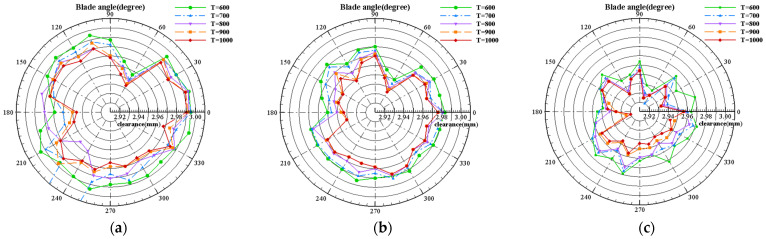
Clearance measurement results at different temperatures: (**a**) rotation speed 1000 r/min; (**b**) rotation speed 2000 r/min; (**c**) rotation speed 3000 r/min.

In order to verify the dynamic measurement ability of the measuring system for the change in blade tip clearance at different temperatures, the experiment temperatures were set to 600 °C, 700 °C, 800 °C, 900 °C, and 1000 °C. Figure 12a–c shows the measurement results of blade tip clearance for the whole rotor at different temperatures when the rotation speed is 1000 r/min, 2000 r/min, and 3000 r/min, respectively.

The following can be seen from Figure 12:

(1) The tip clearance varies significantly with temperature at different rotational speeds. The thermal load increases with the temperature rising, resulting in the increase in blade radial deformation and the decrease in blade tip clearance.
(2) Comparing Figure 12a–c, the centrifugal load increases with the rotation speed increasing, resulting in an increase in blade radial deformation and a decrease in blade clearance.

The measurement results of blade tip clearance corresponding to different temperatures at different rotation speeds are listed in Table 8. The rotor average clearance variations with the temperature rising from 600 °C to 1000 °C are 0.016 mm, 0.014 mm, and 0.017 mm, respectively, when the rotation speed is 1000 r/min, 2000 r/min, and 3000 r/min. It proves that the blade deformation caused by thermal load is similar under different rotation speeds, and the dynamic resolution is proved up to 10 μm by comparing the measured clearance values at different temperatures.

## 4. Discussion

By establishing a high-temperature blade tip clearance measurement platform, calibration and dynamic experiments at high temperatures were carried out to verify the measurement performance of the inductive system, as well as the influence of temperature and rotation speed on the rotor radial deformation. The conclusions are as follows:

(1) The inductive blade tip clearance measurement system has a good dynamic resolution. In a 1000 °C high-temperature environment, with a measurement range up to 3 mm and resolution up to 10 μm, it can detect the blade tip clearance variation under different speed and temperature conditions.
(2) The dynamic clearance measurement results at a certain temperature and rotor speed, respectively, proved that the measuring system can distinguish the rotor radial deformation caused by the thermal load and centrifugal load, and the dynamic resolution is about 10 μm.

The research provides an effective measurement method for engine health monitoring and active clearance control technology. The results indicated that the inductive method based on a high-temperature-resistant and high-resolution sensor has certain advantages compared with other existing methods in terms of range, resolution, and temperature resistance. The long-term temperature resistance of the current inductive sensor without cooling reaches 1000 °C, exceeding most other types of clearance sensors, like microwave sensors and optical sensors, which can withstand temperatures not exceeding 1000 °C. It has a larger range and better resolution (better than 10 μm at 4 mm sensing range) when the probe size is similar to other types of probes. Moreover, the induction sensitivity and temperature resistance can be further enhanced by improving the coil structure and processing technology, adapting to the future advanced turbine environment.

## Figures and Tables

**Table 1:** Comparison of the tip clearance measurement methods [11].

Performance	Discharge Probe Method	Optic Fiber Method	Capacitive Method	Inductive Method	Microwave Method
Temperature, °C	1500	1100	1400	1400	1200
Precision, μm	25	10	15	10	25
Range, mm	6	10	3	5	6
Rotate speed, r/min	8000	60,000	30,000	10,000	18,000
Advantage	High-temperature resistance; not affected by the top surface structure	High precision; compact structure	High-temperature resistance and pressure-resistant; corrosion resistance	Anti-pollution; measuring outside the case	High-temperature resistance and pressure-resistant; corrosion resistance
Disadvantage	Minimum clearance only	Easily contaminated	Influenced by the environment	Large probe structure	High product cost

**Table 2:** Material properties of high-temperature nickel-based alloys.

Material	Melting Point/°C	Coefficient of Thermal Expansion/(10^−6^/°C)	Other Characteristics
Inconel600	1370–1425	15.7	High strength at 650 °C; highest operating temperature in the air is 1175 °C.
Inconel625	1290–1350	16.6	Good tensile properties and fatigue properties below 980 °C.
**Inconel718**	**1210–1344**	**16.2**	Easy processing; high tensile strength and fatigue strength at 700 °C; high oxidation resistance at 1000 °C.
Inconel750	1396–1430	15.8	Good corrosion resistance and oxidation resistance below 980 °C.

**Table 3:** Main performance parameters of the heating furnace.

Parameter	Limited Temperature	Heating Rate	Control Accuracy	Temperature Distribution Difference in Section
Value	1500 °C	<20 °C/min	≤±2 °C	≤±3 °C

**Table 4:** Main parameters of high-temperature dynamic test platform.

Parameter	Value
Rotating speed, r/min	1000–15,000
Driving power, kW	10
Heating temperature, °C	room temperature ~1500
Heating rate, °C/min	≤20
Heating control accuracy, °C	≤±2
Furnace chamber size, mm	120 × 120 × 120
Sampling frequency, MHz	0.1–100
Sampling channel	4 channels
Atmosphere	high temperature and oxidation

**Table 5:** Experimental conditions of verifying the measurement system performance.

Validation Performance	Experiment Operating Parameters			
Dynamic resolution	Temperature, °C	Rotate speed, r/min		
	1000	3000		
Dynamic response	Temperature, °C	Rotate speed, r/min		
	600–1000	1000	2000	3000

**Table 6:** Blade tip clearance measurement result at 1000 °C and 3000 r/min.

Blade No.	Clearance (mm)	Blade No.	Clearance (mm)	Blade No.	Clearance (mm)
1	2.956	9	2.932	17	2.946
2	2.933	10	2.956	18	2.956
3	2.941	11	2.956	19	2.944
4	2.948	12	2.944	20	2.946
5	2.930	13	2.935	21	2.941
6	2.925	14	2.920	22	2.943
7	2.954	15	2.956	23	2.948
8	2.946	16	2.955	24	2.943

**Table 7:** Measurement results of tip clearance with variable rotation speed (unit: mm).

Temperature, °C	Rotate Speed, r/min	Max.	Min.	Avg.	Range
600	1000	2.996	2.956	2.987	0.040
	2000	2.985	2.950	2.976	0.035
	3000	2.979	2.936	2.961	0.043
800	1000	2.995	2.948	2.977	0.047
	2000	2.981	2.940	2.967	0.041
	3000	2.972	2.928	2.952	0.044
1000	1000	2.992	2.943	2.971	0.049
	2000	2.978	2.935	2.962	0.043
	3000	2.956	2.920	2.944	0.036

**Table 8:** Measurement results of tip clearance with variable temperature (unit: mm).

Rotate Speed, r/min	Temperature, °C	Max.	Min.	Avg.	Range
1000	600	2.996	2.956	2.987	0.040
	700	2.995	2.950	2.979	0.045
	800	2.995	2.948	2.977	0.047
	900	2.992	2.945	2.974	0.047
	1000	2.992	2.943	2.971	0.049
2000	600	2.985	2.950	2.976	0.035
	700	2.983	2.945	2.972	0.038
	800	2.981	2.940	2.967	0.041
	900	2.978	2.938	2.963	0.040
	1000	2.978	2.935	2.962	0.043
3000	600	2.979	2.936	2.961	0.043
	700	2.976	2.920	2.955	0.056
	800	2.972	2.928	2.952	0.044
	900	2.958	2.925	2.946	0.038
	1000	2.956	2.920	2.944	0.036

## Data Availability

The data presented in this study are available upon request from the corresponding author.
